# Environmental Factors Associated with *Cryptosporidium* and *Giardia*

**DOI:** 10.3390/pathogens12030420

**Published:** 2023-03-07

**Authors:** Xihan Wang, Xu Wang, Jianping Cao

**Affiliations:** 1 Chinese Center for Tropical Diseases Research, School of Global Health, Shanghai Jiao Tong University School of Medicine, Shanghai 200025, China; 2 Chinese Center for Disease Control and Prevention (Chinese Center for Tropical Diseases Research), National Institute of Parasitic Diseases, Shanghai 200025, China; 3 Key Laboratory of Parasite and Vector Biology, National Health Commission of the People’s Republic of China, Shanghai 200025, China; 4 World Health Organization Collaborating Center for Tropical Diseases, Shanghai 200025, China; 5 One Health Center, Shanghai Jiao Tong University-The University of Edinburgh, Shanghai 200025, China

**Keywords:** One Health, *Cryptosporidium*, *Giardia*, environmental factor, climate, soil

## Abstract

Environmental factors significantly influence the transmission of intestinal protozoan diseases. Cryptosporidiosis and giardiasis are important zoonotic diseases characterized by diarrhea, and are mainly water or foodborne diseases caused by fecal-borne oocysts. The One Health approach effectively addresses environmentally influenced zoonotic diseases. However, the impact of environmental factors on the survival of *Cryptosporidium/Giardia* (oo)cysts or disease transmission is mostly uncharacterized. Associations between cryptosporidiosis and giardiasis incidence and environmental variables (e.g., climatic conditions, soil characteristics, and water characteristics) have been reported; however, the identified relationships are not consistently reported. Whether these are country-specific or global observations is unclear. Herein, we review the evidence for the influence of environmental factors on *Cryptosporidium/Giardia* and corresponding diseases from three perspectives: climatic, soil, and water characteristics. The (oo)cyst concentration or survival of *Cryptosporidium/Giardia* and the incidence of corresponding diseases are related to environmental variables. The associations identified varied among studies and have different levels of importance and lag times in different locations. This review summarizes the influence of relevant environmental factors on *Cryptosporidium/Giardia* from the One Health perspective and provides recommendations for future research, monitoring, and response.

## 1. Introduction

Intestinal protozoan parasites can spread through contaminated water, soil, and food [1], causing zoonotic intestinal diseases. The main symptom is diarrhea, which in severe cases can be life threatening. Cryptosporidiosis and giardiasis are common and significant intestinal parasitic diseases. Waterborne outbreaks or foodborne contamination resulting from contamination by infectious spores or oocysts excreted from host feces are frequent worldwide. They have become the major source of intestinal parasitic diseases globally [2]. Therefore, there is considerable interest in combating cryptosporidiosis and giardiasis.

One Health studies human, animal, and environmental health from a systemic perspective and achieves optimal health outcomes by collaborative efforts across multiple disciplines or sectors [3]. The environmental perspective is an indispensable part of the One Health theory, which is closely related to animal health and human health. Environmental factors affect the transmission of intestinal parasitic diseases [4]. They affect the survival of *Cryptosporidium/Giardia* (oo)cysts and the spread of their corresponding diseases. Therefore, it is also important to understand the role played by environmental factors.

However, among existing studies on *Cryptosporidium* or *Giardia*, there are very few studies on the influence or mechanism of environmental factors, and most of them only explore pathogenesis. Environmental factors have seldom been considered comprehensively. In epidemiological studies, there are few investigations on the specific impact of environmental factors, most of which are limited to a single level or region, such as climate and temperature. There is a lack of comprehensive and systematic understanding of the correlation between *Cryptosporidium*/*Giardia* and environmental factors. To better cope with cryptosporidiosis and giardiasis, we should adopt a One Health approach, which solves complex health problems from a holistic perspective of human–animal–environment health and promotes optimal resource allocation. It is crucial to clarify the role played by environmental factors.

This review aims to summarize the effect of relevant environmental factors on the (oo)cysts of *Cryptosporidium/Giardia* and their corresponding diseases. The analysis is conducted in three aspects: climate, water, and soil. The analysis might help to mitigate the threat posed by *Cryptosporidium* and *Giardia* by providing a framework for risk assessment and disease control.

## 2. Materials and Methods

This review was conducted by searching with keywords in CNKI, PubMed, Scopus, ScienceDirect, and other databases up to 24 January 2022. The main keywords used in this review included: concentration, coverage, climate, climatic, cryptosporidiosis, *Cryptosporidium*, dissolved oxygen, density, ecology, ecological, enteric parasites, environment, environmental factors, extreme weather events, *Giardia*, giardiasis, hardness, humidity, incidence, intestinal protozoan parasites, land, notification, (oo)cysts, pH value, porosity, precipitation, rainfall, runoff, soil, solar radiation, temperature, turbidity, ultraviolet, UV, vegetation, waterborne diseases, water discharge, water level, weather, wind speed, zoonosis, and zoonotic diseases. Firstly, the titles and abstracts of the articles identified in the retrieval process were screened for their appropriateness. Secondly, the full text of articles meeting the selection criteria was extracted for full-text screening after the title and abstract were evaluated. Thirdly, the studies listed in the literature references were reviewed for further evidence and citations.

Initially, the number of articles obtained using all keywords on all platforms was 17,334 (the total number of articles searching for *Cryptosporidium* and related factors was 6034, the total number of articles searching for *Giardia* and related factors was 4498, and the total number of articles searching for intestinal parasites and related factors was 6802). After screening the articles according to the abstract, title, and keywords, and removing the duplicated and irrelevant literature, 162 articles were obtained. After reading the full texts, 51 articles were selected as the main evidence for this review. There have been few studies on environmental factors and the influence of *Cryptosporidium* or *Giardia*; therefore, we tried our best to retain the relevant literature in the screening process.

We applied the following inclusion criteria: (1) The study aimed to investigate the association/mechanism of influence of an environmental factor(s) with *Cryptosporidium*/*Giardia*; (2) it included the relevant environmental variables mentioned in the keywords; (3) the study provided a definitive test/experimental method; and (4) it did not study pathogenesis or was a case report. Articles that did not meet these criteria were excluded from further consideration. We included papers that met the basic standard requirements and did not exclude some papers based on risk of bias. We excluded duplicated and irrelevant papers.

Table 1 summarizes the relevant studies used in the review and the published research findings on the relationship between environmental factors and *Cryptosporidium*/*Giardia*.

## 3. Results

### 3.1. Association between Climatic Factors and Cryptosporidium/Giardia

Climatic factors such as temperature (e.g., mean, maximum, or minimum temperatures over a period of time, usually one month), rainfall, humidity, extreme weather conditions, and solar radiation might affect the life cycles of *Cryptosporidium* and *Giardia* and the spread or incidence of disease. Notably, water temperature is usually unified as a temperature variable instead of a water characteristics in studies.

Temperature and *Cryptosporidium*: Temperature might be positively associated with the survival and density of *Cryptosporidium* oocysts and the incidence of cryptosporidiosis, which peaked during the hottest months, especially in areas with temperate climates [5,6,7,8,9,10,11,12,45,46]. The relationship showed a time lag effect [6,9,11,13]. The time lag could be defined as the time between climatic events and the incidence of diseases [47]. Gonzalez-Moreno et al. [14] reported a positive correlation between the maximum temperature and the number of *Cryptosporidium* oocysts (odds ratio (OR) = 11.46, 95% confidence interval (CI) = 2.70~48.81) in Tanzania. Ma et al. [12] showed positive relationships between the incidence of cryptosporidiosis and the fall temperature (Spearman’s ρ = 0.75) or average temperature (Spearman’s ρ = 0.85) in the wettest season after conducting studies in six Northern/Arctic countries. In England, monthly cryptosporidiosis rates and temperature in the previous month from August to November were positively linked (95% CI = 1.24–2.93, *t*-value = 4.85) [5], which was similar to the situation in India (mean monthly minimum temperature: Rho (Spearman’s rank order correlation coefficient) = 0.608, *p* = 0.003, mean monthly maximum temperature: Rho = 0.475, *p* < 0.001) [7] and New Zealand (average temperature: β = 0.130, SE = 0.060, *p* < 0.01) [10]. Hu et al. [8] confirmed that the incidence of cryptosporidiosis in the database (without zero incidences) correlated positively with temperature (r = 0.349, *p* = 0.000) in Australia. The incidence of cryptosporidiosis was positively associated with monthly maximum temperature at lags of up to 3 months (β = 0.259, *p* < 0.001 in a time series Poisson regression model, β = 0.394, *p* = 0.019 in a seasonal autoregressive integrated moving average (SARIMA) model) in Australia [6].

By contrast, King et al. [15] illustrated that temperature was negatively linked to the survival and infectivity of oocysts shed into the environment. The *Cryptosporidium* oocyst concentration was negatively associated with temperature in Canada [16]. Higher temperatures increased the mortality of *Cryptosporidium* oocysts in America [17]. The mean maximum monthly temperature lagged by 3 months and cryptosporidiosis notifications in rural Australia were negatively related after unadjusted univariate analysis (incidence rate ratio (IRR) = 0.92, 95% CI = 0.88–0.97), but not in urban areas [13]. In New Zealand, average annual temperatures correlated negatively with cryptosporidiosis (source model: IRR = 0.98, 95% CI = 0.977–0.986, distribution model: IRR = 0.98, 95% CI = 0.975–0.984) [18]. Colston et al. [19] showed a skewed, inverted U-shaped relationship between temperature and *Cryptosporidium* when the association was adjusted using multivariable model predictions. Low temperature increased the possibility of the presence or absence of parasites in Canada (air temperature: *p* = 0.041) [20].

Temperature and *Giardia*: Britton et al. [18] reported that average annual temperatures correlated weakly and positively with giardiasis (distribution model: IRR = 1.004, 95% CI = 1.0001–1.007) in New Zealand. Wilkes et al. [16] claimed that *Giardia* cysts were negatively associated with temperature (except in winter) in Canada, which was similar to the findings of Li et al. [21] and Masina et al. (air temperature: *p* = 0.041) [20]. Higher temperature accelerated the die-off of *Giardia* cysts [17]. Chhetri et al. [22] and Lal et al. [10] believed that there was no association between temperature and *Giardia*.

Rainfall: Rainfall might be linked to *Cryptosporidium* [11,12,14,18,45,48,49,50] and *Giardia* [22,49,50], especially after extreme weather events [22,23,24,48]. These effects might involve a time lag [5,9]. Young et al. [50] revealed that extreme weather events and excessive rainfall increased the (oo)cyst concentration, which was supported by Atherholt et al. [23] and Lal et al. [49]. Keeley and Faulkner [25] demonstrated that the *Cryptosporidium* concentration was positively related to rainfall (χ^2^ = 30.6, *p* < 0.0001) in America. Rainfall and the *Giardia* concentration were weakly and positively associated (r = 0.3046, *p* = 0.0334 (1 day); r = 0.3543, *p* = 0.0157 (3 days)) in France [26]. Chhetri et al. [22] claimed that extreme precipitation with a 4–6 week lag was positively associated with reported cryptosporidiosis and Giardiasis cases, which was similar to the findings of Britton et al. (source model: IRR = 1.01, 95% CI = 1.005–1.014; distribution model: IRR = 1.01, 95% CI = 1.004–1.013) in New Zealand [18]. Lal et al. [48] concluded that the peak of cryptosporidiosis was linked to heavy rain. Liu et al. [46] speculated that precipitation might be positively related to the incidence of cryptosporidiosis in China. Gonzalez-Moreno et al. [14] found that precipitation increased the infection rate of *Cryptosporidium* (rainfall (40–80 mm^3^): OR = 5.50, 95% CI = 1.02~29.67; rainfall (>80 mm^3^): OR = 3.12, 95% CI = 1.43–6.85) in Tanzania.

By contrast, Li et al. [21] reported that cumulative precipitation was negatively associated with *Cryptosporidium* oocyst concentration and had a similar relationship with *Giardia* (coefficient = −0.02, 95% CI = −0.03 to −0.006) in central California [27]. Daniels et al. [28] maintained that rainfall could either flush the (oo)cysts into the pond through surface currents or dilute their concentration by flooding or filling the pond. They found that the effect of rainfall on *Giardia* or *Cryptosporidium* (oo)cysts might be positive or negative on different occasions, which was supported by Keeley and Faulkner [25]. In equatorial regions (0–20°) and subtropical regions (20–35°), rainfall correlated positively with the risk of cryptosporidiosis (in equatorial region, rainfall lag of one month: IR = 1.62, 95% CI = 1.042.54, rainfall in February: IR = 2.91, 95% CI = 1.25–6.74; in the subtropical region, rainfall in October: IR = 6.10, 95% CI = 1.02–26.84), while in temperate regions (above 35°), its correlation was negative (in temperate regions, rainfall in December: IR = 0.09, 95% CI = 0.01–0.53), rainfall in January: IR = 0.66, 95% CI = 0.44–0.99 [51]. Lake et al. [5] found that the incidence of cryptosporidiosis correlated negatively with the precipitation of the previous month from August to November (95% CI = −0.0135 to −0.00218, *t*-value = −2.71) in England and Wales. Brune et al. [29] found an inverse association between precipitation and Giardiasis that occurrence 4 weeks later in Canada (OR = 0.6, 95% CI = 0.37–0.97, *p* = 0.038), while Lake et al. [30] reported no association between rainfall and *Cryptosporidium/Giardia* in New Zealand.

Humidity: Some scholars found that the numbers of (oo)cysts increased in warm and moist places, while others believed that only dry or low humidity conditions help spread (oo)cysts. Jagai et al. [45] demonstrated that the relationship between humidity and *Cryptosporidium/Giardia* was similar to that of rainfall, and (oo)cysts could survive more easily in humid areas, which was supported by the results of Wang et al. [52]. By contrast, Colston et al. [19] identified that *Giardia* correlated slightly and negatively with relative humidity. Hu et al. [9] suggested that the incidence of cryptosporidiosis increased at temperatures above 31 °C and relative humidity below 63%. Liu et al. [46] concluded that there was a negative association between humidity and cryptosporidiosis in India, which was different to that in China. In diarrhea samples, the positive detection rate of Cryptosporidium was negatively linked to relative humidity during hotter and drier weather in Delhi, India (mean monthly relative humidity at 8:30 a.m.: Rho = −0.487, *p* = 0.003) [7].

Others: Although some researchers studied the use of artificial ultraviolet (UV) light for oocyst disinfection [31,53], few have studied solar radiation. Solar radiation affects the survival of (oo)cysts in the environment [54]. The viability of *Cryptosporidium* oocysts in seawater decreased by about 90% after a 3-day exposure to solar radiation [32]. King et al. [33] demonstrated that solar UV affected the infectivity of *C. parvum* oocysts in water, being a major driver of oocyst inactivation. Colston et al. [19] found a slight inverse association between solar radiation and *Cryptosporidium/Giardia*. *G. muris* and *C. parvum* (oo)cysts were completely non-infective after batch solar disinfection [34]. Heat from the sun and solar UV worked together to destroy the parasites [35]. In terms of wind speed, Hu et al. [9] found a negative correlation between wind speed and the incidence of cryptosporidiosis, with a lag of 4 weeks (RR = 0.92, 95% CI = 0.90–0.95) in Australia. The concentration of *Cryptosporidium* was negatively associated with the 30 day mean wind speed [21]. Colston et al. [19] found no significant relationship between wind speed and protozoa in general.

### 3.2. Association between Soil Characteristics and Cryptosporidium/Giardia

Soil characteristics are related to *Cryptosporidium* and *Giardia*, although little data is available. Vegetation cover effectively reduces the *Cryptosporidium* oocysts transfer into water, which depends on vegetation residual dry matter, land slope, or runoff [21]. Alum et al. [17] found a higher porosity of the surface caused higher die-off rate of (oo)cysts, and soil particles or organic matter provided protection to the parasite from environmental factors. Soil type and texture affects *Cryptosporidium* inactivation significantly and soil with 49% sand, 27% silt, and 24% clay resulted in rapid inactivation compared with that in soil with 7% sand, 55% silt, and 38% clay at 4 or 35 °C [36]. High temperatures and desiccation in soil increased the die-off rate of *Cryptosporidium*, although the oocyst concentration did not decrease [37]. Colston et al. [19] found that soil moisture increased the risk of *Cryptosporidium* detection (RR = 1.36, 95% CI = 1.25–1.47). The likelihood of detecting *Cryptosporidium* oocysts decreased with increasing soil pH and *Giardia* might be less likely to be detected in areas with shrubs or bare soil than in areas with grazing grass [38]. They reported that vegetated land reduced the rate of water evaporation, and higher soil moisture increased the likelihood of detecting *Giardia* cysts, which might be consistent with the findings of Hu et al. [39]. However, Brown et al. [40] found that *Cryptosporidium* oocysts thrived better in alkaline pH. Vegetation buffer zones significantly reduced the number of *Giardia* cysts in the runoff after a storm [27]. Davies et al. [41] claimed that land slope affected the oocyst concentration in the runoff.

### 3.3. Association between Water Features and Cryptosporidium/Giardia

The evidence for the influence of water characteristics is insufficient, but still enlightening. The density of (oo)cysts correlates positively with water discharge in general; however, if the flow is minimal, the concentration cannot be diluted sufficiently [16]. Lake et al. [5] concluded that the incidence of cryptosporidiosis was positively linked to the current month’s maximum river flow (95% CI = 0.0445–0.119, *t*-value = 4.29) in England, with other factors considered. However, Brankston et al. [11] indicated that average water flow and human cryptosporidiosis were weakly and negatively associated (OR = 0.93, 95% CI = 0.870.99, *p* = 0.04) in Canada. Brune et al. [29] found an inverse association between *Giardia* and the water level that lagged by 1 month (IRR = 0.1, 95% CI = 0.01–0.85, *p* < 0.05) in Canada. *Giardia* cyst concentration was positively related to the turbidity and pH value of water [21]. A similar association was found between the density of *Cryptosporidium* and water turbidity [50]. Dissolved oxygen was related to *Giardia* cyst concentration, while the pH value, water hardness, and river flow were related to the *Cryptosporidium* oocyst concentration, and turbidity was related to both [23]. Salinity plays a significant role in *Giardia*: compared with that in dark seawater (97.1 h, T99), cysts survived longer in canal water (121.3 h, T99) [32].

## 4. Discussion

Environmental factors (including climate, soil, and water characteristics in this review) influence the survival/infectiveness of *Cryptosporidium/Giardia* (oo)cysts or the incidence of cryptosporidiosis/giardiasis. Various studies have come to different conclusions. Whether environmental factors become risk or protective factors depends on multiple factors and the mechanisms are complex. Although some previous studies have investigated the influencing factors of cryptosporidiosis/giardiasis, the attention paid to the environmental perspective remains insufficient. Most reviews only consider one perspective, especially climatic factors, while studies on soil or water characteristics are relatively rare. Despite this, we can still extract useful evidence from the existing literature to support this review. Environmental factors influence the survival and infectivity of *Cryptosporidium* and *Giardia* (oo)cysts or the incidence of corresponding diseases. Next, the correlation between environmental factors and *Cryptosporidium*/*Giardia* as well as possible causes/mechanisms will be analyzed from three aspects: climatic, water, and soil characteristics.

Climatic characteristics: Factors such as temperature, rainfall, humidity, wind speed, and solar radiation play important roles, and they can be considered as risk or protective factors for *Cryptosporidium* or *Giardia*. High temperatures can improve the survival of *Cryptosporidium/Giardia* (oo)cysts and promote disease progression to some extent, although conversely, high temperatures increase the inactivation of (oo)cysts. *Cryptosporidium* oocysts were more likely to survive in warm and wet weather [52], while temperature inactivation is a key abiotic factor affecting (oo)cyst survival and infectivity in the environment [11,15]. Temperature might increase the risk of infection by promoting pathogen infectivity, shedding in animal hosts, and increasing the interactions between pathogens and hosts [55]. Rainfall and extreme weather events are associated with the (oo)cyst concentration, which depends on the situation. The correlation is positive because excessive rainfall and runoff might mobilize oocysts [11,22,42,48], which then leads to an increase in microbial infections [43]. More intense precipitation events cause more sediment disturbance and resuspension of infectious oocysts. Meanwhile, this might result in oocyst sedimentation due to increased turbulence and binding of organic matter in water [56]. Drought caused an increase in concentration of pathogens in water sources, which could be washed out by subsequent rainfall [10]. Li et al. [21] demonstrated that the *Cryptosporidium* oocyst concentration correlated negatively with precipitation, because precipitation did not bring more oocysts into the water, but rather diluted the concentration of oocysts already present in the sampled surface water. Studies have shown positive and negative associations between relative humidity and *Giardia*/*Cryptosporidium*, while wind speed and solar radiation were mainly negatively associated with both.

Soil characteristics: Relevant factors include the slope, vegetation cover, soil moisture, porosity, pH value, soil type, and texture. Soil moisture can increase the survival of oocysts and affect disease transmission, while dry soil partially inhibits the survival of oocysts [37,38]. Vegetated land has lower concentrations of oocysts than bare land, and vegetated buffers effectively retain fine *Cryptosporidium* oocysts deposited in cattle manure, thus minimizing potential contamination of water sources by infected oocysts [27,44]. Inactivation of oocysts in the soil depends to a large extent on soil temperature, but to a lesser extent on soil texture, and precipitation can mobilize oocysts in the soil [36,56]. Studies of other land features have not provided definitive conclusions, which also vary among studies.

Water characteristics: The main factors involved are runoff, dissolved oxygen, water pH, the water level, and turbidity. Among them, runoff has been studied more extensively. When water flow conditions are insufficient to dilute or flush pathogens from waterways, it can be assumed that pathogens will concentrate. Conversely, high flow rates might dilute or flush pathogens from waterways. When runoff was extremely low and oocyst concentrations could not be diluted, the risk of disease increased [16]. Other water features are less studied and further investigation of their mechanisms of action is required.

It is worth mentioning that the time lag can be defined as the interval between climatic events and the incidence of diseases [47]. Reasons for the time lag effect of environmental factors might be related to differences in transmission routes, incubation periods, time differences in seeking medical services, the amplification of infection because of interpersonal transmission, or survival of the (oo)cysts in the environment [6,11,57].

By summarizing the three aspects, we found that environmental factors play a significant role in *Cryptosporidium*/*Giardia* transmission and the mechanism is complex. However, the number of relevant studies is still insufficient and more evidence-based studies are needed in the future. Nevertheless, in the following text, we will try to analyze the external or internal factors that may affect the environmental factors in their role.

There is no doubt that environmental factors are also influenced by other factors. They do not act individually. Transmission of *Cryptosporidium* and *Giardia* is a complex process with multiple hosts and pathways that vary greatly within and between countries. The differences in results among studies are linked to other factors, such as social factors or geographical differences, suggesting multifactorial effects. We summarized three aspects.

Firstly, different locations (latitude and longitude) or climate type (which is related to geographical location) affect the role of environmental factors. Jagai et al. [45] indicated that temperature was positively associated with cryptosporidiosis in moist tropical climates, while there was no association between them in arid or semiarid areas. Liu et al. [46] suggested that seasonal patterns varied by geography. Although humidity might be a strong seasonal driver of cryptosporidiosis in humid tropical climates, the incidence of cryptosporidiosis in more temperate northern regions correlated inversely with humidity. Meanwhile, climate patterns are related to latitude and longitude. Lal et al. [51] claimed that rainfall played a different role in regions with different latitudes, such as equatorial, subtropical, or temperate latitudes. The association between the incidence of cryptosporidiosis and maximum river flow would be positive only when precipitation and temperature were considered [5], suggesting that a less direct mechanism was at work. Ajjampur et al. [7] claimed that depending on the climate category, rainfall was more important in the tropics, while temperature was more important in temperate climates. Propagation patterns in tropical countries might differ from those in temperate countries, resulting in different seasonal patterns. Thus, geographical location plays an important role among environmental factors, confirming the complexity of the influencing mechanism.

Secondly, various environmental factors influence each other. Atherholt et al. [23] demonstrated that rainfall affected turbidity, river flow, and other water characteristics, thus affecting the oocyst concentration. If the flow velocity is high enough, particulate matter and particulate-related microbes can be directly affected by the resuspension of river bottom sediments. Rainfall increases the flow/velocity of water or the concentration of dissolved oxygen, and reduces the pH, alkalinity, or hardness of water, which correlate with the (oo)cyst concentration. Consistently, Young et al. [50] stated that the effect of rainfall on (oo)cysts might be influenced by water turbidity, flow rate, and sediment. Chhetri et al. [22] found that extreme precipitation increased turbidity directly or increased surface runoff into water systems indirectly, thereby increasing pathogen transfer (e.g., allowing animal waste to enter surface water). Heavy rain could wash animal and human waste into surface sources of drinking water, contaminate water sources, and increase the risk of disease [24]. Davies et al. [41] concluded that the oocyst load in runoff was significantly influenced by factors such as vegetation condition, soil slope, and rainfall intensity/duration. During rainfall, the short time between the onset of rainfall/runoff in wet or saturated soils caused high runoff volumes, facilitating the transport of (oo)cysts in the environment [58]. Therefore, there are complex correlations among environmental factors.

Finally, other factors, such as human activities or economic conditions, work with environmental factors. Lal et al. [51] concluded that lower latitudes were more likely to have higher population densities and lower socio-economic development, leading to a higher risk of disease by means other than environmental transmissions, such as poor sanitation, water, and sanitation infrastructure, which was supported by Lal [59] and Tegen et al. [60]. Differences in the prevalence of human cryptosporidiosis might be related to poor sanitation and the lack of health education and good health habits [46,61]. Agricultural practices, recreational water use, and other human activities affect the infection rate, together with environmental factors [48]. Kent et al. [13] suggested that the relationship between temperature and cryptosporidiosis was reversed in urban and rural Australia; however, the reason was unclear. It might be related to economic conditions, human activities, and health conditions. Forbes et al. [24] claimed that the greater the proportion of land with pristine habitat conditions, the greater the risk of cryptosporidiosis. Anthropogenic land-use changes (e.g., deforestation, fragmentation, and agricultural development) and human alteration of wildlife habitats increase the risk of zoonotic transmission. Land use is associated with the risk of detection of *Cryptosporidium* oocysts in soil, with a higher risk in areas around barn cleaners and agricultural fields compared with that in unused land [38]. Human activities, such as land use, agricultural practices, and recreational water use, influence the effect of environmental factors [25,48,49]. In addition, microorganism interactions, such as those associated with *Escherichia coli*, have an effect [23,26,62]. Studies need to consider these other external factors.

Therefore, the role played by environmental factors in the survival of *Cryptosporidium/Giardia* (oo)cysts and the transmission of giardiasis/cryptosporidiosis is complex and the interconnections among the environmental factors cannot be separated from each other. In addition, environmental factors are influenced by other external factors that must be considered. Importantly, the interactions that occur between these factors have synergistic or antagonistic effects; although how they affect the survival of (oo)cysts and disease transmission is largely unknown. More attention needs to be paid to them in future studies.

This review has some limitations. Firstly, the studies cited might have reporting and publication biases. Secondly, relevant data and studies are insufficient. Few scholars study the environmental factors related to *Cryptosporidium/Giardia*, with most of them focusing on climatic factors, especially temperature and rainfall. Studies on soil or water variables and other climatic variables are insufficient.

## 5. Conclusions

This review summarizes the associations between environmental factors and *Cryptosporidium/Giardia* (in terms of disease transmission, incidence, oocyst concentration, or survival). Although environmental factors and *Giardia*/*Cryptosporidium* are associated, the relative contribution of each parameter varies worldwide. This review considered various environmental factors related to climate, soil, and water, allowing integration of the influences of environmental factors in multiple dimensions, in contrast to previous reviews that mainly considered single perspectives.

Environmental factors are governed by multiple factors with complex mechanisms when affecting the occurrence of cryptosporidiosis/giardiasis or the survival of (oo)cysts. Firstly, different locations (latitude and longitude) or climate type (which is related to geographical location) affect the role of environmental factors. Secondly, various environmental factors influence each other (such as rainfall (climatic factors) and turbidity/river flow of water (water characteristics)). Thirdly, other factors, such as human activities or economic conditions, interact with environmental factors. These internal and external factors cannot be ignored in analyzing the complexity of environmental factors. More research and evidence are needed to verify such complex mechanisms, and more epidemiological and experimental studies should be performed in the near future.

Nevertheless, we identified environmental factors that play a significant role. The One Health approach is a practical way to tackle zoonotic diseases [63]. Climate change and irreversible changes in land and water bodies caused by human activities mean that the incidence of these diseases and the concentration or survival of their (oo)cysts could increase, thereby augmenting the disease burden. Therefore, it is crucial to adopt a One Health approach and to consider health from the human–animal–environment perspective. In recent studies, the effects of environment factors on *Cryptosporidium/Giardia* have received insufficient attention. This review provides suggestions for the authorities to develop strategies and measures against parasitic diseases from an environmental standpoint. We provide further evidence that environmental changes represent challenges for zoonotic disease management. This review might help with the implementation and execution of the One Health approach in the future.

## Figures and Tables

**Table 1:** The relevant studies used in the review.

Reference	Study Period	Location	Sample Size	Analytical Method	Association Found
[5]	1989–1996	England and Wales (Europe)	Over 52,000 (exclude 1999 cases who had recent foreign travel)	Ordinary least-squares regression	A positive association between monthly cryptosporidiosis rates and temperature in the previous month from August to November. A negative association between monthly cryptosporidiosis rates and precipitation in the previous month from August to November. A positive association between monthly cryptosporidiosis rates and maximum river flow in the current month between April and July. A positive association between monthly cryptosporidiosis rates and maximum river flow in the current month between August and November, only when temperature and precipitation in the previous month were included in the model.
[6]	1996–2004	Australia	NA	Time series Poisson regression and seasonal auto-regression integrated moving average (SARIMA) models	A positive association between maximum temperature at lags of 1 to 3 months and *Cryptosporidium*. A positive association between relative humidity at a lag of 1 month and *Cryptosporidium*.
[7]	2005–2008	India (Asia)	2579	Mann–Whitney U test and Fisher’s exact test	A positive association between temperature and *Cryptosporidium*. A negative association between humidity and *Cryptosporidium*. A higher rate of cryptosporidiosis positivity during hotter and drier weather especially in Delhi.
[8]	2001	Australia	NA	Three-stage spatiotemporal classification and regression tree (CART) models	A positive association between temperature and the incidence of cryptosporidiosis for the database without zero incidences. No association between temperature and the incidence of cryptosporidiosis for the full database in any season. A positive association between rainfall and *Cryptosporidium*.
[9]	1996–2004	Australia	NA	Time series zero-inflated Poisson (ZIP) and classification and regression tree (CART) models Cross-correlation function	A negative association between rainfall at lags of 0–1 week and cryptosporidiosis incidence. A negative association between wind speed at a lag of 4 weeks and cryptosporidiosis incidence. A positive association between maximum temperature at lags of 0–8 weeks and cryptosporidiosis incidence. The incidence of cryptosporidiosis might increase at temperatures above 31 °C and relative humidity below 63%, otherwise the effect on cryptosporidiosis was insignificant
[10]	1997–2008	New Zealand (Oceania)	8092 cases of cryptosporidiosis and 10,424 cases of giardiasis	Seasonal Auto Regressive Integrated Moving Average (SARIMA) models	No climatic factors were significantly associated with giardiasis. Cryptosporidiosis was positively associated with the average temperature of the previous month.
[11]	Human: 2009–2015, Bovine: 2008–2014	Canada (North America)	214 bovine and 87 human cases	Logistic regression models and a case-crossover approach	A positive association between the maximum ambient air temperature at 0, 9, or 14 days and human cryptosporidiosis. No association between rainfall and the incidence of human cryptosporidiosis in this study. A negative association between average water flow and the incidence of human cryptosporidiosis.
[12]	2004–2016 (cryptosporidiosis)	Six Northern/Arctic countries or country parts	NA	Spearman’s correlation coefficient and stepwise regression	A positive association between the incidence of cryptosporidiosis and autumn temperature. A positive association between the incidence of cryptosporidiosis and mean temperature of the wettest quarter. A positive association between the incidence of cryptosporidiosis and annual maximum monthly precipitation.
[13]	2001 to 2009	Australia	NA	A negative binomial regression model	A positive association between the mean maximum monthly temperature lagged by 2 months and cryptosporidiosis notifications in metropolitan areas. A positive association between the mean minimum monthly temperature and cryptosporidiosis notifications in metropolitan areas. A negative relationship between the mean maximum monthly temperature lagged by 3 months and cryptosporidiosis notifications in rural areas. A negative relationship between the mean minimum monthly temperature lagged by 3 months and cryptosporidiosis notifications in rural areas. No association was found with rainfall for any area examined.
[14]	2001–2003, 2008–2010	Tanzania (Africa)	406	Multivariate logistic regression analysis	A positive association between rainfall and *Cryptosporidium*. A positive association between maximum temperature and *Cryptosporidium*.
[15]	Water samples: 2003	Australia	NA	Liner regression	Temperature is a critical parameter in the survival and infectivity of oocysts shed into the environment. Higher temperatures decrease autonomous survival and infectivity.
[16]	2004–2008	Canada (North America)	1171	CART (Classification and Regression Tree) and binary logistical regression techniques Spearman’s rank correlation	*Cryptosporidium* and *Giardia* oocyst and cyst densities were positively associated with surface water discharge, and negatively associated with air/water temperature during spring–summer–fall. Some of the highest *Cryptosporidium* oocyst densities were found to be associated with low discharge conditions on small stream orders, suggesting wildlife as a contributing fecal source.
[17]	NA	America (North America)	NA	Multivariable regression analysis	A positive association between temperature and die-off of *Cryptosporidium*. A positive association between temperature and die-off of *Giardia*. The direct correlation between higher die-off rates of parasites (oo)cysts and higher porosity of surfaces.
[18]	1997 to 2006	New Zealand (Oceania)	NA	Negative binomial regression	A negative association between average annual temperatures and cryptosporidiosis in both the source model and distribution mode. A weakly positive association between average annual temperatures and giardiasis that only reached borderline significance in the distribution model. A positive association between rainfall and giardiasis/cryptosporidiosis (both models). Urban/rural status was a strong predictor of cryptosporidiosis in both the univariate and multivariate regression analyses.
[19]	NA	Data from 19 countries in Central and South America, Sub-Saharan Africa and South and Southeast Asia	NA	An IPD-MA framework, modified Poisson models with robust variance estimation, generalized linear models (GLMs)	No association between relative humidity and *Cryptosporidium*. A slight inverse association between relative humidity and *Giardia*. A positive association between soil moisture and *Cryptosporidium*. A slight inverse association between solar radiation and *Cryptosporidium* or *Giardia*. The adjusted association between temperature and *Cryptosporidium* was a skewed, inverted U-shaped relationship, while the association with *Giardia* took on a more sinusoidal shape.
[20]	2016	Canada (North America)	55 water samples	Exact logistic regressions	Cumulative precipitation, water level, and turbidity were not associated with the presence/absence of parasites. Low water/air temperature increased the possibility of the presence/absence of parasites.
[21]	2012–2013	Central California (North America)	NA	Logistic regression, Poisson regression	*Cryptosporidium* oocyst concentrations were negatively associated with 30-day mean wind speed and cumulative precipitation. *Giardia* spp. cyst concentrations were positively associated with turbidity and the pH of water, and negatively associated with 24 h mean air temperature.
[22]	1997–2009	Canada (North America)	7422	Poisson regression and a distributed lag nonlinear regression model (DLNM)	No association between temperature and cryptosporidiosis cases was reported. No association between temperature and giardiasis cases was reported. A positive association between extreme precipitation with a lag of 4–6 weeks and cryptosporidiosis & giardiasis.
[23]	1996	America (North America)	NA	Spearman rank correlations	A positive association between extreme weather events and *Giardia* cysts. A significant correlation of *Giardia* and *Cryptosporidium* concentrations with turbidity levels. *Cryptosporidium* oocyst concentrations were associated with 12 other parameters, 9 of which also were associated with *Giardia*. Of the associated parameters, the ones that had the highest correlations with parasite concentrations were coliphage, DO, air, and water temperature (*Giardia* only), total and fecal coliforms, *E. coli*, alkalinity, hardness, daily average pH, river flow (*Cryptosporidium* only), and turbidity (both parasites).
[24]	2001–2018	Australia	NA	A Bayesian spatio-temporal analysis	A positive association between heavy/extreme rainfall and *Cryptosporidium*.
[25]	2000–2002	America (North America)	193	Kruskal–Wallis non-parametric test, a time series analysis (PROC ARIMA)	A positive association between rainfall and *Cryptosporidium*. Although mean *Giardia* cyst peaks occasionally coincided with rainfall peaks, the lowest monthly rainfall occurred in March of the first year when cyst levels were lowest. The sample cross-correlation functions revealed a peak in positive correlation with rainfall at lag zero, with negative correlation near the 2 month time lag, forward and backward.
[26]	2005–2007	Paris in France (Europe)	162	The nonparametric Spearman’s rho, the nonparametric Wilcoxon test	A weak and positive association between rainfall and *Giardia*. No association between rainfall and *Cryptosporidium*.
[27]	2002–2004	California (North America)	350	A negative binomial regression model	Vegetation buffer zones significantly reduced *Giardia* cysts in storm runoff. A negative association between cumulative precipitation and the concentration of *Giardia*.
[28]	2012–2013	India (Asia)	NA	Separate multivariable models of protozoa contamination	Rainfall sometimes increased the *Giardia* concentration and sometimes diluted it.
[29]	2006–2013	Canada (North America)	403 cases	A Poisson multivariable regression model	An inverse association between *Giardia* and water level lagged by 1 month. An inverse association between within-stratum highest precipitation levels occurring 4 weeks prior to human cases and giardiasis occurrence in the truncated time series.
[30]	1997–2005	New Zealand (Oceania)	NA	Time-series studies, ordinary least-squares regression	In the summer and autumn, the cryptosporidiosis rate was positively associated with temperature in the current and previous month. No association between rainfall and cryptosporidiosis.
[31]	NA	America (North America)	NA	Linear regression (t- and F-tests)	The use of artificial ultraviolet (UV) light for oocyst disinfection.
[32]	NA	Hawaii (North America)	NA	Linear regression	A 90% reduction was identified in the viability of *Cryptosporidium* in marine waters after a 3 day exposure to solar inactivation. *Giardia* survived longer in canal water than in dark seawater, indicating that salinity has a greater effect on its inactivation than water quality.
[33]	2005–2006	Australia	NA	Linear regression	Solar UV can rapidly inactivate *C. parvum* in environmental waters.
[34]	NA	NA	1 × 10^7^ purified *C. parvum* oocysts or 5 × 10^5^ of *G. muris* cysts	A test of comparison of proportion, a pair-wise multiple comparison procedure and one-way ANOVA	Results showed that cysts of *G. muris* and oocysts of *C. parvum* are rendered completely noninfective after batch SODIS exposures of 4 and 10 h, respectively, and is also likely to be effective against waterborne cysts of *Giardia lamblia*.
[35]	2007	Goromonzi in Zimbabwe (Africa)	NA	NA: Control experiment (the average results with their standard deviations)	There is a synergistic effect between the heat and the UV light produced by the sun on the inactivation of *Giardia*.
[36]	NA	Soil samples: Sydney (Australia)	NA	Fluorescence in situ hybridization (FISH) and the Student–Newman–Keuls Test	Soil type emerged as a significantly influential factor for *Cryptosporidium* inactivation.
[37]	NA	Water samples: The State of Israel (Asia)	NA	NA: Control experiment	The die-off of *C. parvum* in saturated and dry loamy soil was monitored over time using immunofluorescence assay (IFA) and PCR to estimate oocysts viability and by cell culture to estimate oocysts infectivity.
[38]	NA	Southeastern New York State (North America)	782	Logistic regression	A negative association between the pH of the soil and the likelihood of detecting *Cryptosporidium* oocysts. Vegetation at the sampling site was significantly associated with the risk of detecting *Giardia* in the soil, and areas that were brush or bare soil were less likely to test positive for *Giardia* than land that had managed grass. A positive association between soil moisture and *Giardia* cysts.
[39]	NA	Western Australia	NA	NA: sludge storage trials and small scale soil amendment trials	A positive association between soil moisture and *Giardia* cysts.
[40]	NA	NA	NA	NA: control experiment	*Cryptosporidium* oocysts thrive better in soils with alkaline pH than in those with acidic pH.
[41]	NA	Australia	NA	Analysis of variance and analysis of covariance with the SAS generalized linear model procedure simulation experiment	Land slope was an important factor affecting the concentration of oocysts in runoff.
[42]	2006–2007	New York (North America)	NA	Spearman rank statistical test	A positive relationship between the occurrence of *Cryptosporidium* oocysts and extreme weather events. A positive relationship between the occurrence of *Giardia* cysts and extreme weather events.
[43]	15 months	Germany (Europe)	NA	NA: control experiment	A risk of water-related infections through water-bound activities exists, especially after rain events and in times when thunderstorms are common.
[44]	2002–2004	America (North America)	NA	A negative binomial regression model	Vegetation cover can effectively reduce the amount of *Cryptosporidium* transfer from terrestrial manure to water.

## Data Availability

Not applicable.
